# Prognostic value of perineural invasion in prostate needle biopsies: a population-based study of patients treated by radical prostatectomy

**DOI:** 10.1136/jclinpath-2019-206300

**Published:** 2020-02-07

**Authors:** Peter Ström, Tobias Nordström, Brett Delahunt, Hema Samaratunga, Henrik Grönberg, Lars Egevad, Martin Eklund

**Affiliations:** 1 Medical Epidemiology and Biostatistics, Karolinska Institutet, Stockholm, Sweden; 2 Department of Clinical Sciences, Danderyd University Hospital, Stockholm, Sweden; 3 Pathology and Molecular Medicine, Wellington School of Medicine, Wellington, New Zealand; 4 Aquesta Pathology, Brisbane, Queensland, Australia; 5 Department of Oncology, S:t Göran Hospital, Stockholm, Sweden; 6 Department of Oncology-Pathology, Karolinska Institutet, Stockholm, Sweden

**Keywords:** prostate, cancer, surgery

## Abstract

**Aims:**

Despite being one of the major pathways for the spread of malignant tumours, perineural invasion (PNI) has not conclusively been shown to have an independent prognostic value for prostate cancer. Prostatic biopsy constitutes the major pathology workload in prostate cancer and is the foundation for primary treatment decisions and for this reason we aimed to estimate the prognostic value of PNI in biopsies.

**Methods:**

We followed 918 men who underwent radical prostatectomy (RP) from the prospective and population based STHLM3 study until biochemical recurrence with a median follow-up of 4.1 years. To strengthen the evidence, we combined the estimates from the largest studies targeting the prognostic value of PNI in the biopsy. We also estimated the OR of advanced stage as radical prostatectomy for PNI positive and negative men.

**Results:**

The estimated prognostic value based on our data suggested an approximately 50% increased risk of biochemical recurrence if PNI was present in the biopsy (p=0.06). Even though not statistically significant on the 5% level, this estimate is consistent with similar studies, and by combining the estimates there is in fact strong evidence in support of an independent prognostic value of PNI in the biopsy (p<0.0001). There was also an independent increased risk of advanced stage at RP for positive men (OR 1.85, p=0.005).

**Conclusions:**

The evidence supporting a clinically relevant and independent prognostic value of PNI is strong enough to be considered for pathology reporting guidelines.

## Introduction

The perineural space—the potential space between neural axons and their perineural capsule—is one of the major pathways for the spread of malignant tumours.^1^ Perineural invasion (PNI) is broadly defined as malignant cells in, around or through a nerve, implying that tumour cells do not necessarily need to be in the perineural space, but may infiltrate anywhere within the nerve.^2^ It has also been proposed that for PNI to be differentiated from perineural compression in the prostate, at least one-third of the nerve circumference must be involved.^3^

Although PNI is recognised as an avenue for tumour spread beyond the parent organ, it is not well established as a prognostic variable in prostate cancer. In a systematic review and meta-analysis of the prognostic value of PNI, Zhang *et al* concluded that PNI is *probably* associated with shorter biochemical recurrence-free survival.^4^ The included studies were conducted with PNI in both in biopsy and radical prostatectomy (RP) specimen and with following primary treatment by either RP or radiotherapy. Despite the plethora of studies on this topic they did note that the results were not conclusive and concluded that formal controlled trials were needed. Most published studies to date relate to outcomes for PNI detected in RP specimens, while relatively few focus on the prognostic significance of PNI in needle biopsies.^4^ This is unfortunate as biopsies constitute the bulk of the pathology workload and are the basis for the decisions made regarding primary treatment.

Neither the College of American Pathologists structured reporting protocols nor the International Collaboration on Cancer Reporting guidelines require reporting of PNI in the prostate, as it is noted that there is no established evidence of its prognostic value.^5^ Despite this, the European Association of Urology, does recommend that the presence of PNI should be noted in the pathology report.^6^ In view of these conflicting recommendations, the conclusions of the meta-analysis of Zhang *et al* and the observation that PNI is a principal pathway of escape of prostate cancers, there is a need for additional studies to determine the prognostic significance of PNI in needle biopsies for prostate cancer.^1^

In this study we have assessed the prognostic value of PNI in needle biopsies taken from patients whose prostate cancer was detected through screening-by-invitation. This involved the evaluation of needle biopsies with follow-up from men who were diagnosed with prostate cancer in the prospective and population-based STHLM3 trial and who then underwent radical RP. We also combined this with the evidence from the largest prospective studies of the prognostic value of biopsy PNI.

## Methods

### Databases and participants

**Figure 1:**
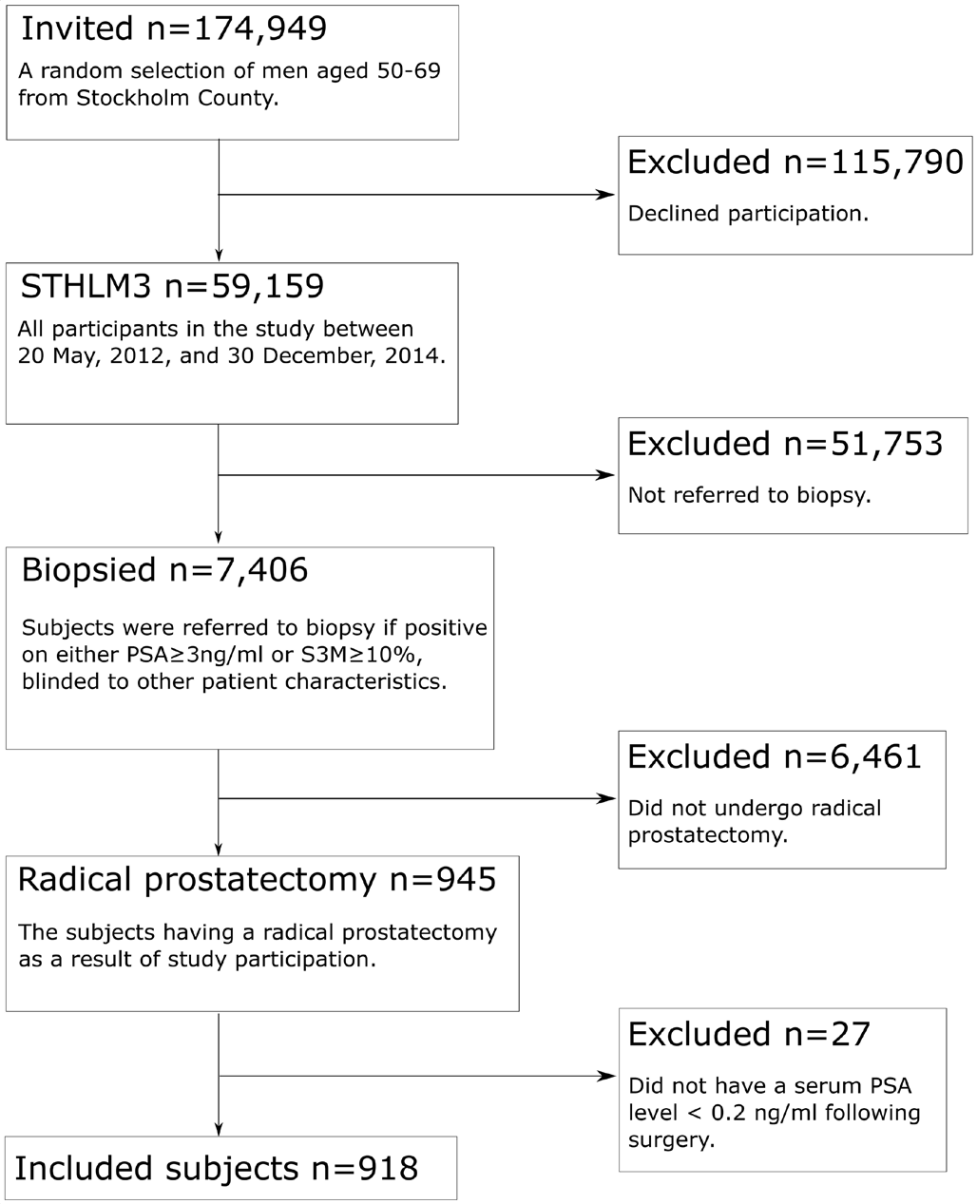
Patient flow diagram.

Prostate cancer screening was evaluated in the prospective and population-based STHLM3 study that was undertaken from 28 May 2012 to 30 December 2014.^7 8^ In this study, invitations to participate were issued to 174 949 men, aged 50–69 years and resident in Stockholm County, Sweden. A total of 59 159 men agreed to take part in the study and 7406 (12.5%), who were shown to have positive results in at least one of two screening tests (PSA ≥3 ng/mL or Stockholm3 risk ≥10%), were referred for biopsy.^7^ The referral was blinded to other clinical information and the assessment was undertaken by a single specialist uro-pathologist (LE), who was blinded to the tests results. Following a histological diagnosis of prostate cancer 945 men underwent RP. Of these men 918, who had a serum PSA level <0.2 ng/mL following surgery, were included in the study cohort (figure 1).

Trial data relating to the 945 men who underwent RP were linked to the Stockholm PSA and Biopsy Register at the Karolinska Institute, Stockholm, which tracks the timing and results of all PSA tests and prostate biopsies undertaken on men resident in Stockholm County.^9^ Time to death was determined from the cause-of-death register held by The Swedish National Board of Health and Welfare, while loss to follow-up due to emigration out of Stockholm County was detected through the Total Population Register of Statistics Sweden.

### Outcome, exposure and follow-up

**Figure 2:**
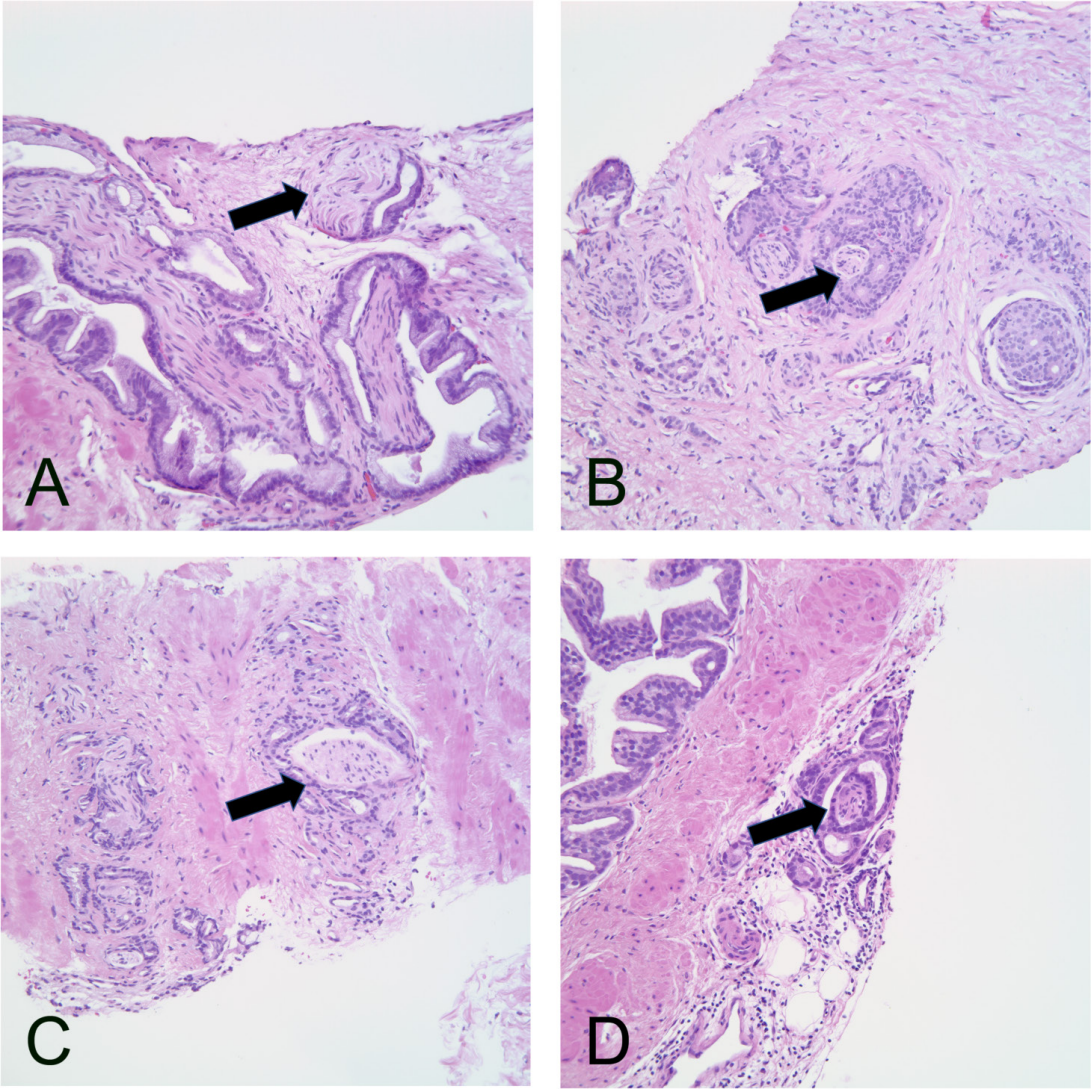
Perineural invasion of prostatic adenocarcinoma. (A) Circumferential cancer growth around large nerves and cancer growing along a small nerve (arrow). (B) Small nerves surrounded by cancer (arrow). (C) Cross-section through nerve (arrow) surrounded by cancer. (D) Small nerve in periprostatic fat, also including ganglion cell (arrow). All microphotographs stained with hematoxylin and eosin (20× lens magnification).

PNI was defined as the presence of tumour infiltrating one or more nerves. PNI was diagnosed when prostatic carcinoma was found immediately adjacent to a nerve, either along the nerve or surrounding it. A nerve was defined as a pale eosinophilic structure with slender, parallel nuclei and fibrillar material in the background. Examples of the study pathologist’s classification of PNI are shown in figure 2. Biochemical recurrence (PSA relapse) was taken as the outcome, being defined by PSA higher than 0.2 ng/mL, as recommended by The American Urological Association and the European Association of Urology.^10 11^ Follow-up commenced from the date of the RP and continued until biochemical recurrence, death, emigration from Stockholm County or the end-of-study interval on 31 December 2018, whichever came first. The time scale in the analysis was time since RP.

### Statistics

To estimate the prognostic value of PNI we used Cox proportional hazard regression. The estimated HR was adjusted for potential confounders. Due to the potential confounding of grade and stage of the cancer and the total tumour burden, we adjusted the analysis for patient age (linear continuous), serum PSA levels at presentation (1–3, 3–5, 5–10 and 10+ ng/mL), digital rectal examination (positive or negative), length of cancer in the core biopsy (linear continuous; the extent measured from end to end) and International society of urological pathology (ISUP) grade (1, 2, 3 and 4–5). All clinical variables were taken from the pathology report of the needle biopsies to address the question of the clinical benefit of accounting for PNI as the primary treatment decision.

In a second model we addressed the question of the prognostic value of biopsy PNI after primary treatment by RP and included pathological stage category (pT2, pT3a, pT3b/T4), based on the eighth edition of the AJCC staging classification, and surgical margin status (positive or negative) in the analysis.^12^

Three of the potential confounders were continuous measures. The model fit for these variables was assessed as categorical (as in table 1), linear and spline effects. The Akaike information criterion (AIC) was used for model selection, and for ties (AIC differences less than 2) we erred on the side of interpretability. No other alternative model specifications were evaluated. In addition, we performed stratified analyses on subjects with ISUP 1–2 and subjects with ISUP 3–5.

**Table 1:** Subject characteristics for the study cohort. The event, biochemical recurrence, was defined as PSA above 0.2 ng/mL and person-years is time from radical prostatectomy to event or censoring

	PNI positive men (n=146)	PNI negative men (n=772)
	No. (%)	No. (%)
Age		
<54	15 (10)	104 (13)
55–59	23 (16)	125 (16)
60–64	53 (36)	203 (26)
≥65	55 (38)	340 (44)
PSA (ng/mL)		
<3	17 (12)	123 (16)
3–5	62 (42)	345 (45)
5–10	45 (31)	222 (29)
≥10	22 (15)	82 (11)
Digital rectal examination		
Normal	89 (61)	643 (83)
Abnormal	57 (39)	129 (17)
Cancer length (mm)		
0–5	6 (4)	201 (26)
5–10	16 (11)	175 (23)
10–20	41 (28)	189 (24)
20–40	47 (32)	152 (20)
>40	36 (25)	55 (7)
ISUP		
1	13 (9)	208 (27)
2	78 (53)	373 (48)
3	34 (23)	106 (14)
4–5	21 (14)	85 (11)
Number of PNI positive cores		
0	0 (0)	772 (100)
1	90 (62)	0 (0)
2	31 (21)	0 (0)
3	11 (8)	0 (0)
4 or more	14 (10)	0 (0)
Pathological stage		
T2	73 (50)	584 (76)
T3a	62 (42)	161 (21)
T3b/T4	11 (8)	27 (3)
Surgical margin		
Negative	113 (77)	603 (78)
Positive	33 (23)	169 (22)
Events	31	82
Person-years	555	3143

All variables were from the biopsy except pathological stage and surgical margin. Pathological stage was confined to the prostate (pT2), extraprostatic growth (pT3a) or seminal vesicle invasion (pT3b); there was only one case of pT4.

PNI, perineural invasion.

We estimated the 5-year adjusted relapse-free survival for PNI positive and negative subjects, each standardised to the cohort covariate distribution.^13^ This makes the 5-year survival comparable and also reflects the population. The underlying model was the model for the HR that did not include variables obtained at the RP since we were primarily interested in primary treatment decision.

As a complement to the prognostic analysis, we estimated the OR for advanced pathological stage defined as pT3 or higher among PNI positive subjects compared with PNI negative subjects, adjusted for the same variables as in the time-to-event analysis.

We used a random-effect meta-analytic model to combine our estimate of the biopsy PNI prognostic HR with the three largest studies on the topic using a similar design to ours.^14–17^ The inclusion criteria were time-to-event studies with biochemical recurrence (PSA higher than 0.2 ng/mL) as outcome from which we could retrieve multivariate adjusted HR, a cohort defined by RP and PNI from biopsy as the exposure. To the best of our knowledge we have included all studies published in English that fulfil these criteria.

There were no statistically significant violations to the proportional hazard assumption for any covariates in each of the two models. Missing data in the study cohort was confined to seven values on pathological stage and four values on surgical margin, which were imputed by the mode. Statistical significance was defined at 5% risk level and all CIs were 95% two sided. The analysis was undertaken using R, V.3.6.0 (R Foundation for Statistical Computing) and the R packages survival for HR, stdReg for standardised survival and metafor for the random-effect model for combining HRs. All codes for this project are available in online (https://github.com/PeterStrom/PNI_clinical).

## Results

**Figure 3:**
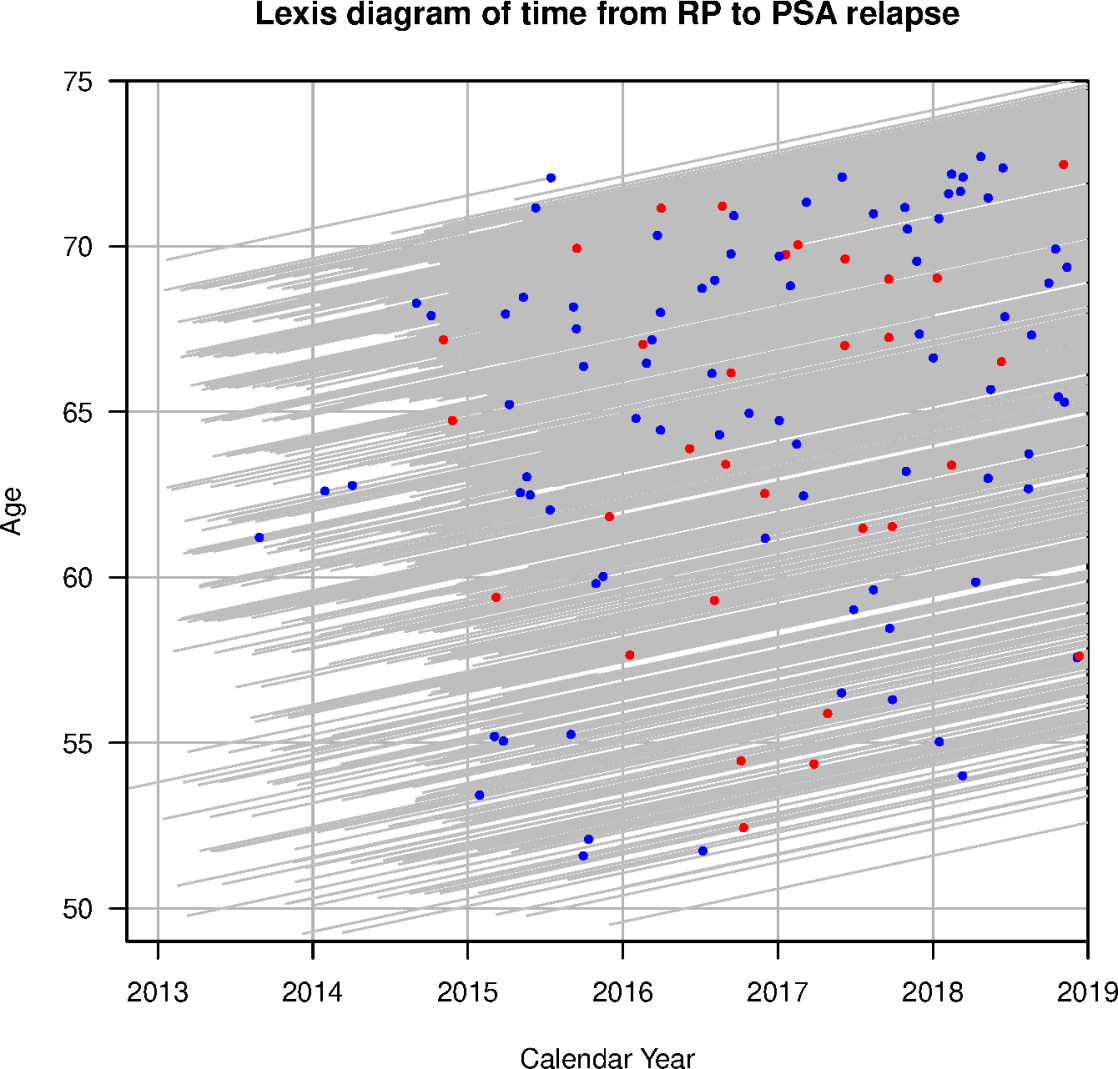
A Lexis diagram. Each grey line is the timeline for a subject in the study. It starts at the date of the RP and end at the event or censoring; events are marked with a dot, blue for men without PNI and red for those with PNI in the biopsy. The y-axis shows the age at the biopsy preceding the RP. RP, radical prostatectomy.

Among the 918 subjects in the study cohort, 146 (16%) were positive for PNI. Biochemical recurrence was seen in 113 patients, with 31 (27%) being in subjects with PNI. The mean and median follow-up intervals were 4.0 and 4.1 years, respectively, with an IQR 1.1 years. The distribution of dates of RP, age and follow-up interval are shown in figure 3. Age and PSA distribution was similar between men with or without PNI; however, the men with PNI were more likely to have a palpable tumour, larger tumour burden and higher grade. When considering the finding from the RP, the two groups were just as likely to have a positive surgical margin, although the PNI group more frequently showed extra-prostatic extension of tumours and seminal vesicle invasion (table 1).

The OR for advanced pathological stage (n=261 positive and n=657 negative) for PNI positive subjects was 1.85 (95% CI 1.20 to 2.83) compared with PNI negative subjects (table 2). This statistically significant (p=0.005) estimate was adjusted for variables from the biopsy assessment.

**Table 2:** Advanced stage at radical prostatectomydefined as pT3 or higher (n=261) versus PT2 (n=657)

	Cases	Controls	OR (95% CI)
Perineural invasion			
Negative	188	584	1.0
Positive	73	73	1.85 (1.20 to 2.83)
Age			
Per 5 year	261	657	1.25 (1.06 to 1.46)
PSA (ng/mL)			
1–3	15	125	1.0
3–5	87	320	2.08 (1.13 to 3.86)
5–10	101	166	4.26 (2.28 to 7.93)
≥10	58	46	5.49 (2.68 to 11.26)
Digital rectal examination			
Negative	171	561	1.0
Positive	90	96	1.70 (1.15 to 2.51)
Cancer length			
Per 10 mm	261	657	1.39 (1.24 to 1.55)
ISUP			
1	37	184	1.0
2	120	331	1.08 (0.69 to 1.70)
3	57	83	1.83 (1.06 to 3.16)
4–5	47	59	1.87 (1.04 to 3.38)

Multivariate adjusted ORs for the variables presented in the table.

**Figure 4:**
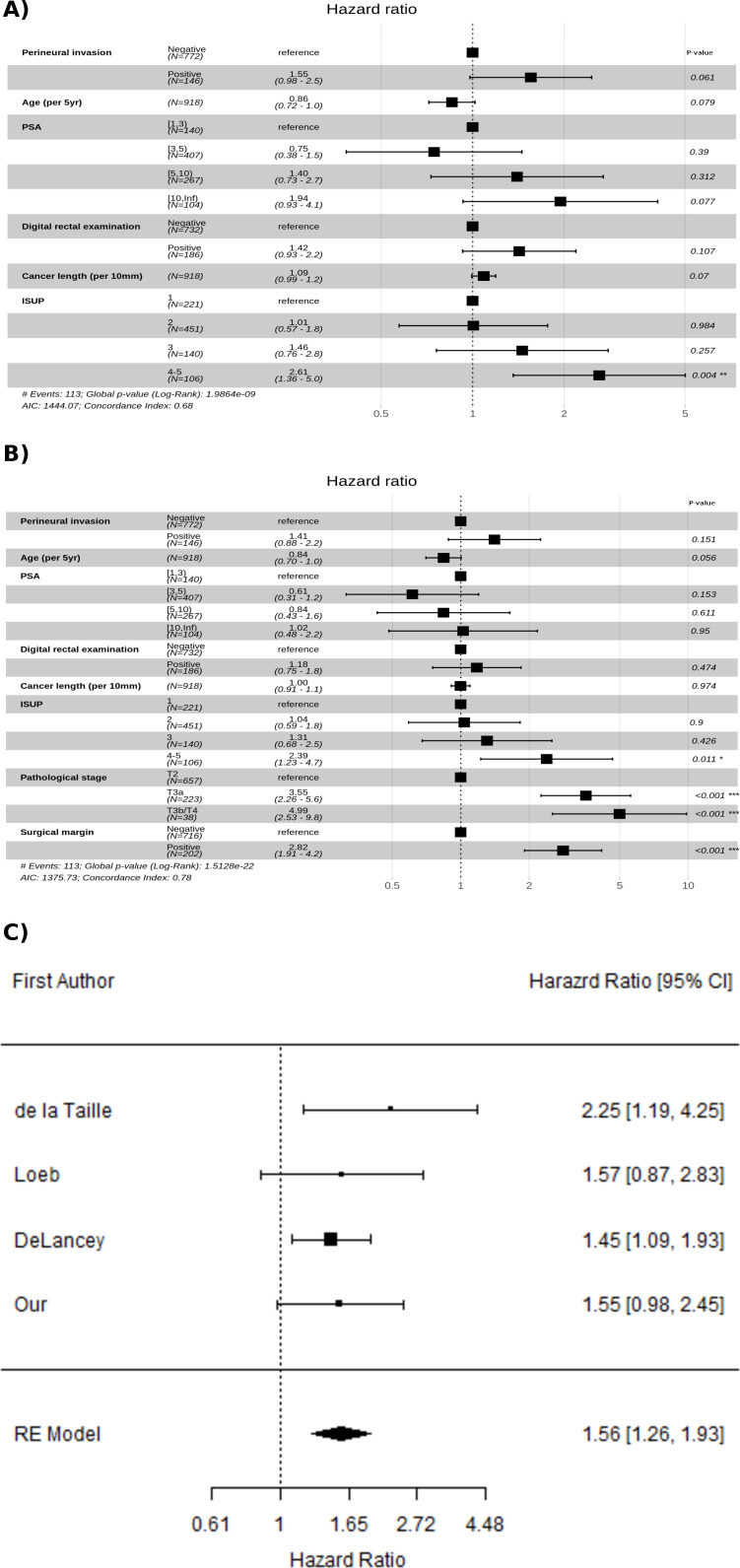
Cox proportional hazard estimated HR for PNI and the adjusting variables on time to biochemical recurrence. A) A model adjusted only for clinical information available at biopsy and B) a model which also includes findings from RP (pathological stage and surgical margins). The event was biochemical recurrence (above 0.2 ng/ml) and censoring at death, emigration and end-of-study 31 December 2018. C)Adjusted estimates of prognostic value of perineural invasion in biopsy from the largest studies including our own, together with a combined estimate using a random-effect (RE) model. RE, Random effects.

The HR for biochemical recurrence-free survival among PNI positive versus negative was 1.55 (95% CI 0.98 to 2.5), p=0.061, adjusted for variables from the biopsy assessment. When findings from the RP were included the estimate was 1.41 (95% CI 0.88 to 2.2), p=0.151 (figure 4). The figure also shows the effects of the adjusting variables on the time to biochemical recurrence. The former model was used to estimate 5-year relapse-free survival standardised to the study participants (a screening-by-invitation cohort). The estimates were 0.79 (95% CI 0.72 to 0.87) for PNI positive and 0.86 (95% CI 0.82 to 0.89) for PNI negative subjects. The former model was also used for stratifying on ISUP 1–2 (events=63, n=672) and ISUP 3–5 (events=50, n=246). This resulted in HR for biopsy PNI of 1.19 (95% CI 0.59 to 2.4), p=0.62 and 1.94 (95% CI 1.02 to 3.67), p=0.04, respectively.

Incorporation of our estimated HR and those from the three largest published studies resulted in an estimated HR 1.56 (95% CI 1.26 to 1.93). We did not observe any heterogeneity between studies (I^2^=0%, p=0.68). The p value for the estimate pooled HR was <0.0001, indicating that the studies together strongly support the prognostic value of PNI in the biopsy.

## Discussion

This study suggests an independent increased risk of around 50% for biochemical recurrence when PNI is detected on biopsy; an estimate that agrees well with previous studies. The estimate was not statistically significant on the 5% level and needs to be evaluated in the context of previous studies. When the RP findings were incorporated the estimated effect remained high. A stratified analysis on ISUP grade suggests that most of the effect of biopsy PNI is found in high grade cases. However, this analysis is based on relatively few events in each stratum and the effect estimates needs to be interpreted in context of the wide confidence intervals. In this study was biopsy PNI independently associated with advanced pathological stage, which agrees with the majority of previous publications.

DeLancey *et al* evaluated the prognostic significance of PNI in biopsies in a large cohort of 3226 patients treated by RP.^17^ In contrast to our study, they adjusted for adverse pathological findings derived from analysis of the RP, which provided information that was not available at the time of decision of initial treatment. Therefore, this analysis may have resulted in a conservative estimate of the prognostic value of PNI since these variables likely form a causal pathway, that is, PNI may lead to adverse findings on RP which, in turn, may confer a poor prognosis. Despite providing limited information, the study provides strong evidence of an approximately 50% increased risk of disease progression for men with PNI identified in the biopsy. Loeb *et al* had earlier conducted a similar study involving 1256 subjects where multivariable adjusted PNI failed to reach statistical significance as an independent predictor of biochemical recurrence.^16^ This study has been used as an argument against a prognostic value of PNI in biopsy; however, it should be noted that non-significance does not imply support for the null hypothesis.^18^ Interestingly, the point estimate for the prognostic value of PNI in the study of Loeb *et al* was slightly higher than reported by DeLancey *et al*, so rather than showing no effect they, in fact, presented weak evidence in support of such an effect. An earlier study by de la Taille *et al* demonstrated an even higher relative risk for poor outcome for biopsies with of PNI (HR: 2.25, 95% CI 1.19 to 4.23).^15^ All of these previous studies, as well as our own, were similar in design, utilised the same definition of biochemical recurrence and demonstrated high point estimates of the prognostic value for PNI, irrespective of whether it reached statistical significance or not. We are not aware of a single study on biopsy PNI with an estimated value close enough to the unity to be clinically non-relevant, and with CI narrow enough to provide reasonable level of support for such a claim.

A combined HR estimate of the four studies discussed above and including our own series, using a random-effect model, was 1.56 (95% CI 1.26 to 1.93). All four studies suggested a similar increase in risk for PNI positive biopsies, and taken together they strongly support an independent prognostic value of PNI in the biopsy. Even though the design and analysis were very similar in all studies (biochemical recurrence >0.2 ng/mL, biopsy PNI, RP cohort, Cox regression), it should be noted that the adjusting variables differed somewhat; the main difference was that DeLancey *et al* also included postsurgical variables in their model. If the research question is focused on the prognostic value of biopsy-related variables alone, then this will likely result in a conservative estimate. This can also be seen in a comparison of the model with and without RP information in the present study.

The strength of our study is that the data were derived from a population-based and prospective controlled trial in a screening-by-invitation setting. Referral of patients for biopsy was blinded except serum PSA and the S3M (multivariable diagnostic prediction model for prostate cancer) test. The cancers in the histological sections were examined by a single pathologist and this included evaluation of PNI. Assessment of all biopsies followed a systematic protocol and the pathologist was blinded to all clinical variables.

The main limitation of our study is that the reporting of the pathological findings in the RP specimens was not performed by a single pathologist. Another limitation is that the PNI status of the patient was known at the time the decision relating to primary treatment was made. This is known as confounding by indication. In this case the effect is most likely marginal as in current clinical practice PNI does not have a strong influence on treatment decisions. If this was a confounding influence, it should arguably result in a more conservative estimate since the information cannot result in less aggressive treatment. This study only concerns men who are candidates for RP, while men undergoing radiotherapy as initial treatment or who were followed with active surveillance were not part of the study population.

The evidence of PNI in prostate biopsies as an independent prognostic marker for prostate cancer is largely considered to be conflicting. The reason for this is likely the large uncertainty attached to the majority of the studies. This screening-by-invitation cohort suggests a substantial prognostic value although associated with some uncertainty. By evaluating the results in the context of similar studies, we can in fact show very strong statistical evidence in favour of an independent prognostic value of PNI in the biopsy. Taking these results into account, it is apparent that there is sufficient evidence to indicate that PNI in prostate biopsies is of clinical relevance.

**Take home messages**

- Perineural invasion (PNI)—cancer growing inside nerves—is one of the primary paths of cancer spread. It has not been shown conclusively to be an independent prognostic marker in prostate biopsies. In this study we follow nearly 1000 men who has done complete surgical removal of the prostate due to prostate cancer and evaluate the impact of PNI on PSA relapse. We also combine the evidence from similar studies and conclude that they individually suggest an approximately 50 per cent increase in risk of relapse if the biopsy contains PNI, and that they together show strong statistical support for this risk estimate.
